# Association of Canonical Wnt/β-Catenin Pathway and Type 2 Diabetes: Genetic Epidemiological Study in Han Chinese

**DOI:** 10.3390/nu7064763

**Published:** 2015-06-15

**Authors:** Jinjin Wang, Jingzhi Zhao, Jianfeng Zhang, Xinping Luo, Kaiping Gao, Ming Zhang, Linlin Li, Chongjian Wang, Dongsheng Hu

**Affiliations:** 1 Department of Traditional Chinese Medicine Prevention, Preventive Medicine Research Evaluation Center, Henan University of Traditional Chinese Medicine, Zhengzhou 450008, China; E-Mail: wangjinjin510@yahoo.com; 2 Department of Epidemiology, Shenzhen University School of Medicine, Shenzhen 518060, China; E-Mails: lxp2005@szu.edu.cn (X.L.); gao_kp@szu.edu.cn (K.G.); zhangming@szu.edu.cn (M.Z.); 3 Military Hospital of Henan Province, Zhengzhou 450000, China; E-Mail: jingzhi_zhao@sina.com; 4 Henan Armed Police Corps Hospital, Zhengzhou 450000, China; E-Mail: jeefzhang106@yahoo.com; 5 Department of Epidemiology, College of Public Health, Zhengzhou University, Zhengzhou 450001, China; E-Mails: lilinlin@zzu.edu.cn (L.L.); jwcj2008@zzu.edu.cn (C.W.)

**Keywords:** WNT signaling pathway, polymorphisms, type 2 diabetes, Chinese

## Abstract

We aimed to investigate the associations of polymorphisms in Canonical Wnt/β-catenin pathway (WNT) signaling genes (including low-density lipoprotein-related protein 5 [*LRP5*] and transcription factor 7-like 2 [*TCF7L2*] gene) and the downstream gene glucagon (*GCG*) and risk of type 2 diabetes mellitus (T2DM) in a Han Chinese population. We genotyped the single nucleotide polymorphisms (SNPs) for *LRP5*, *TCF7L2* and *GCG* gene were genotyped in 1842 patients with T2DM and 7777 normal glucose-tolerant healthy subjects. We used multifactor dimensionality reduction (MDR) and multiplicative logistic regression adjusting for sex, age, anthropometric measurements and lipid levels to investigate the gene-gene interactions for the risk of T2DM. Among the five SNPs in *LRP5*, the recessive model of rs7102273 and the haplotype GCTCC were associated with T2DM risk; the haplotype GCTTC was associated with decreased risk. For *TCF7L2*, the rs11196218 genotype GA and the haplotype CCG, TTG, TTA were associated with T2DM risk; whereas, the haplotype CTG and TCG were associated with decreased risk. Both MDR and multiplicative logistic regression revealed potential gene–gene interactions among *LRP5*, *TCF7L2*, and *GCG* associated with T2DM. The WNT signaling pathway may play a significant role in risk of T2DM in Han Chinese people.

## 1. Introduction

Type 2 diabetes mellitus (T2DM) is a metabolic disorder of multiple etiologies characterized by chronic hyperglycemia with disturbed carbohydrate, fat and protein metabolism. The disease results from progressive insulin secretory defect in a background of insulin resistance and accounts for 90% of diabetes mellitus [1]. The International Diabetes Federation (IDF) estimated that more than 371 million people had diabetes in 2012, and about 4.8 million people died due to diabetes worldwide. In China, the prevalence of T2DM was 9.3% in 2011 and was estimated to increase to 12.1% in 2030 [2]. The incidence and prevalence of T2DM has reached epidemic proportions all over the world. Thus, investigating the etiology of T2DM and intervention measures is urgently needed.

Despite many studies, the mechanisms of T2DM remain uncertain. The Canonical Wnt/β-catenin pathway (WNT) signaling plays a well-established role in the metabolic syndrome, especially T2DM. Recent data puts WNT signaling pathway in a pivotal role in regulating pancreas development as well as islet function, insulin production and secretion [3,4,5,6]. In addition, there seems to be another indirect link between WNT signaling pathway and T2DM: The classical WNT signaling pathway can regulate the transcription of the proglucagon gene in order to leading the GLP-1 expression. Furthermore, the intracellular effect of GLP-1 on pancreatic beta-cells appears to be mediated partly by the classical WNT signaling pathway [7]. The WNT signaling pathway is composed of Wnts, secreted antagonists, seven transmembrane cell surface receptors (Frizzled) and co-receptors (e.g., LRP5), and beta-catenin. Ligand binding to both the Frizzled and LRP coreceptor can activate GSK-3, and lead to beta-catenin coactivating transcription factors (e.g., T-Cell Factor, TCF) to regulate the downstream proglucagon gene (e.g., *GCG*) resulting to GLP-1 expression [8]. Thus, the key effectors of the WNT signaling pathway include low-density lipoprotein receptor-related protein 5 (*LRP5*), transcription factor 7-like 2 (*TCF7L2*), and the downstream gene glucagon (*GCG*). *LRP5* was found to play an important role in glucose and lipid metabolism in animal studies [9] and was located in the IDDM4 region in the long arm of chromosome 11, linked to type 1 diabetes [10]. *TCF7L2* was found to be the strongest candidate associated gene with T2DM [11]. *GCG* expressed in intestinal epithelial endocrine L-cells located on chromosome 2q24.2, encoded several proteins crucial for regulation of proglucagon and glucagon-like peptide (GLP)-1 and -2. One of the most important functions of GLP-1 is as an incretin hormone. GLP-1 plays a crucial role in the development and treatment of T2DM [12].

From the functions of these three genes in regulating glucagon secretion, we hypothesized that the WNT signal pathway genes are associated with T2DM. Therefore, we aimed to confirm an association of SNPs in *LRP5*, *TCF7L2*, and *GCG* and T2DM in Han Chinese. We used tag SNP to screen candidate SNPs for *LRP5* and *GCG* because of few studies of these genes. For *TCF7L2*, we selected the SNP confirmed in previous studies.

## 2. Results

### 2.1. Characteristic of Study Participants

In all, 925 males of 1842 T2DM patients selected were males; 3214 of 7777 controls were males. Compared with controls, diabetic patients had significantly greater anthropometric and metabolic values (all *p* < 0.001) (Table 1).

**Table 1:** Characteristics of study participants.

Characteristics	Cases (*n* = 1842)	Controls (*n* = 7777)	*p*
Sex			
Male	925 (50.20)	3214 (41.32)	<0.001
Female	917 (49.80)	4563 (58.68)	
Age (years)	54 (20–85)	49 (25–75)	<0.001
Body mass index (kg/m^2^)	27.38 (18.51–50.45)	23.83 (18.51–43.50)	<0.001
Waist circumference (cm)	90.50 (60.60–200.00)	81.35 (58.00–155.00)	<0.001
SBP (mmHg)	128.00 (87.67–218.67)	121.00 (81.33–218.33)	<0.001
DBP (mmHg)	81.33 (44.00–136.00)	77.00 (49.00–140.00)	<0.001
Fasting plasma glucose (mmoL/L)	7.63 (3.45–27.13)	5.19 (3.23–6.09)	<0.001
HDL-C (mmoL/L)	1.10 (0.55–4.73)	1.14 (0.47–2.48)	<0.001
LDL-C (mmoL/L)	2.94 (0.20–10.47)	2.40 (−1.70–8.80)	<0.001
TG (mmoL/L)	1.37 (0.38–14.28)	1.32 (0.33–11.06)	<0.001
TC (mmoL/L)	4.86 (1.58–13.66)	4.28 (1.66–10.23)	<0.001

Data are number (%) or median (range).

### 2.2. Association of LRP5 and T2DM

Among SNPs for *LRP5*, the frequency of only rs7102273 significantly differed between cases and controls (*p* = 0.005) (Table 2). On logistic regression analysis, the recessive and the dominant models for rs7102273 were associated with risk of T2DM (OR = 1.381, 95% CI = 1.121–1.701, *p* = 0.002; OR = 1.128, 95% CI = 1.010–1.260, *p* = 0.003, respectively). After adjusting for confounders such as sex, age, anthropometric measurements, and metabolic measurements, only the recessive model was associated (OR = 1.274, 95%CI = 1.009–1.609, *p* = 0.001) (Table 3). The haplotype GCTCC was associated with increased disease risk and GCTTC with decreased risk (OR = 1.182, 95% CI = 1.031–1.354, *p* = 0.001; OR = 0.701, 95% CI = 0.576–0.853, *p* = 0.001, respectively) (Table 4). The population-attributable risk proportion (PARP) for the haplotype GCTCC was 1.22%.

**Table 2:** Genotypic and allelic distributions of single nucleotide polymorphisms in LRP5 gene among Han Chinese in China.

SNPs	Cases	Controls	*p*
rs3758644			
AA	1 (0.01)	1 (0.05)	0.341
GA	58 (3.15)	213 (2.74)	
GG	1783 (96.84)	7563 (97.21)	
A	60 (1.63)	215 (1.38)	0.257
G	3624 (98.37)	15,339 (98.62)	
rs7102273			
CC	140 (7.60)	469 (6.04)	0.005
TC	593 (32.19)	2750 (35.35)	
TT	1109 (60.21)	4558 (58.61)	
C	873 (23.70)	3688 (23.71)	0.986
T	2811 (76.30)	11,866 (76.29)	
rs4930588			
GG	48 (2.61)	170 (2.19)	0.515
TG	438 (23.78)	1825 (23.47)	
TT	1356 (73.61)	5782 (74.34)	
G	534 (14.50))	2165 (13.92)	0.365
T	3150 (85.50))	13,389 (86.08)	
rs12363572			
TT	12 (0.65)	58 (0.75)	0.150
CT	225 (12.22)	1080 (13.89)	
CC	1605 (87.13)	6639 (85.36)	
T	249 (6.76)	1196 (7.69)	0.054
C	3435 (93.24)	14,358 (92.31)	
rs11228303			
TT	10 (0.55)	26 (0.34)	0.236
CT	215 (11.83)	916 (12.01)	
CC	1592 (87.62)	6687 (87.65)	
T	235 (6.47)	968 (6.34)	0.786
C	3399 (93.53)	14,290 (93.66)	

Data are number (%).

**Table 3:** Associations of SNPs in *LRP5* gene and T2DM in Han Chinese.

SNPs	Genotypes	Unadjusted ORs (95% CI)	*p*	Adjusted ORs * (95% CI)	p *
rs3758644	GG	1		1	
	AA + GA *vs.* GG	1.168 (0.871–1.566)	0.300	1.091 (0.784–1.518)	0.607
rs7102273	TT	1		1	
	CT	0.880 (0.766–1.010)	0.069	0.908 (0.780–1.056)	0.211
	CC	1.220 (0.968–1.539)	0.092	1.157 (0.894–1.496)	0.268
	CC *vs.* TC + TT	1.381 (1.121–1.701)	0.002	1.274 (1.009 –1.609)	0.042
	CC + TC *vs.* TT	1.128 (1.010–1.260)	0.033	1.066 (0.943–1.204)	0.307
rs4930588	TT	1		1	
	TG	1.064 (0.917–1.235)	0.412	1.098 (0.932–1.293)	0.263
	GG	1.133 (0.800–1.605)	0.482	1.128 (0.768–1.658)	0.539
	GG *vs.* TG + TT	1.176 (0.840–1.648)	0.344	1.103 (0.759–1.604)	0.607
	GG + TG *vs.* TT	1.023 (0.908–1.154)	0.706	1.063 (0.931–1.214)	0.368
rs12363572	CC	1		1	
	CT	0.879 (0.744–1.038)	0.127	0.940 (0.783–1.128)	0.504
	TT	0.860 (0.457–1.620)	0.641	0.861 (0.421–1.762)	0.682
	TT *vs.* CT + CC	0.993 (0.525–1.880)	0.983	0.932 (0.454–1.911)	0.847
	TT + CT *vs.* CC	0.862 (0.739–1.005)	0.058	0.926 (0.782–1.096)	0.371
rs11228303	CC	1	-	1	-
	CT	0.986 (0.841–1.155)	0.858	0.951 (0.798–1.134)	0.606
	TT	1.685 (0.810–3.506)	0.163	1.724 (0.760–3.912)	0.193
	TT *vs.* CT + CC	1.639 (0.778–3.449)	0.193	1.766 (0.768–4.062)	0.181
	TT + CT *vs.* CC	0.986 (0.842–1.155)	0.860	0.951 (0.798–1.134)	0.577

* Adjusted for sex, age, anthropometric measurements, TC, TG, HDL-C, and LDL-C.

**Table 4:** Associations of haplotypes of SNPs in *LRP5* gene and T2DM.

	Haplotypes					Cases	Controls	ORs (95% CI)	*p*
	rs3758644	rs7102273	rs4930588	rs12363572	rs11228303				
1	A	T	T	C	C	52 (1.44)	182 (1.20)	1.209 (0.887–1.649)	0.229
2	G	C	G	C	C	364 (10.07)	1558 (10.26)	0.981 (0.870–1.106)	0.754
3	G	C	G	C	T	35 (0.97)	107 (0.70)	1.389 (0.947–2.036)	0.091
4	G	C	G	T	C	15 (0.41)	63 (0.41)	1.014 (0.579–1.778)	0.960
5	G	C	T	C	C	286 (7.90)	1031 (6.78)	1.182 (1.031–1.354)	0.016
6	G	C	T	C	T	23 (0.64)	82 (0.54)	1.162 (0.729–1.852)	0.528
7	G	C	T	T	C	120 (3.32)	712 (4.68)	0.701 (0.576–0.853)	0.001
8	G	C	T	T	T	8 (0.22)	51 (0.34)	0.667 (0.325–1.414)	0.297
9	G	T	G	C	C	101 (2.79)	365 (2.40)	1.175 (0.940–1.469)	0.156
10	G	T	T	C	C	2362 (65.28)	10,047 (66.10)	0.967 (0.896–1.044)	0.387
11	G	T	T	C	T	157 (4.34)	662 (4.36)	0.996 (0.833–1.190)	0.963
12	G	T	T	T	C	88 (2.43)	310 (2.04)	1.193 (0.938–1.517)	0.149
13	G	T	T	T	T	7 (0.19)	29 (0.19)	1.087 (0.482–2.450)	0.840

### 2.3. Association of TCF7L2 and T2DM

Cases and controls differed in genotype distribution (*p* = 0.001) but not allelic distribution of rs11196218 (*p* = 0.308) (Table 5). The GA genotype and the recessive model of rs11196218 were associated with increased risk of T2DM (OR = 1.435, 95% CI = 1.141–1.805, *p* = 0.002; OR = 1.319, 95% CI = 1.057–1.646, *p* = 0.014, respectively). After adjustment for confounders such as sex, age, anthropometric measurements, and metabolic measurements, only the GA genotype remained associated with increased disease risk (OR = 1.344, 95% CI = 1.044–1.730, *p* = 0.001) (Table 6).

The haplotype analysis involved combining our previous results for rs7903146 and rs290487 (Wang *et al.* [13]) and current results for rs11196218 with *TCF7L2*. We found eight haplotypes involving rs7903146, rs290487, and rs11196218 in *TCF7L2* among study participants. Hap-analysis revealed the haplotypes CCG, TTA, and TTG were associated with increased T2DM risk (OR = 1.130, 95% CI = 1.040–1.228, *p* = 0.004; OR = 1.392, 95% CI = 1.156–1.676, *p* = 0.001; OR = 1.442, 95% CI = 1.029–2.020, *p* = 0.002, respectively) and CTG and TCG associated with decreased disease risk (OR = 0.873, 95% CI = 0.811–0.939, *p* = 0.001; OR = 0.747, 95% CI = 0.565–0.988, *p* = 0.001, respectively) (Table 7). The PARP for genotype GA of rs11196218 was 11.73% and for haplotypes CCG, TTG and TTA was 2.96%, 1.20%, and 1.35%.

**Table 5:** Genotypic and allelic distributions of rs11196218 in *TCF7L2* gene among Han Chinese.

SNPs	Cases	Controls	*p*
rs11196218			
AA	98 (5.44)	540 (7.05)	0.001
GA	771 (42.76)	2961 (38.64)	
GG	934 (51.80)	4163 (54.31)	
A	967 (26.82)	4041 (26.37)	0.579
G	2639 (73.18)	11,287 (73.63)	

Data are number (%).

**Table 6:** Association of rs11196218 in *TCF7L2* gene and T2DM in Han Chinese.

SNPs	Genotypes	Unadjusted ORs (95% CI)	*p*	Adjusted ORs * (95% CI)	p *
rs11196218	AA	1		1	
	GA	1.435 (1.141–1.805)	0.002	1.344 (1.044–1.730)	0.022
	GG	1.236 (0.986–1.551)	0.067	1.202 (0.937–1.542)	0.148
	GG *vs.* GA + AA	0.904 (0.816–1.002)	0.054	0.930 (0.830–1.042)	0.212
	GG + GA *vs.* AA	1.319 (1.057–1.646)	0.014	1.261 (0.989–1.609)	0.062

* Adjusted for sex, age, anthropometric measurements, TC, TG, HDL-C, and LDL-C.

**Table 7:** Associations of haplotypes of SNPs in *TCF7L2* gene and T2DM.

	Haplotypes			Cases	Controls	ORs (95% CI)	*p*
	rs7903146	rs290487	rs11196218				
1	C	C	A	308 (8.54)	1262 (8.23)	1.040 (0.913–1.185)	0.552
2	C	C	G	928 (25.72)	3595 (23.46)	1.130 (1.040–1.228)	0.004
3	C	T	A	583 (16.16)	2514 (16.40)	0.982 (0.890–1.084)	0.723
4	C	T	G	1499 (41.55)	6884 (44.91)	0.873 (0.811–0.939)	0.001
5	T	C	A	31 (0.86)	130 (0.85)	1.007 (0.679–1.494)	0.970
6	T	C	G	59 (1.63)	333 (2.17)	0.747 (0.565–0.988)	0.041
7	T	T	A	46 (1.27)	135 (0.88)	1.442 (1.029–2.020)	0.032
8	T	T	G	154 (4.27)	475 (3.10)	1.392 (1.156–1.676)	0.001

Data are number (%).

### 2.4. Association of GCG and T2DM

Cases and controls did not differ in genotype and allelic distributions for rs12104705 (*p* > 0.05) (Table 8). We found no association of genotypes of rs12104705 and T2DM (see Table 9).

**Table 8:** Genotypic and allelic distributions of rs12104705 in *GCG* gene among Han Chinese.

SNPs	Cases	Controls	*p*
rs12104705			
TT	9 (0.49)	46 (0.60)	0.875
CT	239 (13.26)	1021 (13.32)	
CC	1555 (86.25)	6597 (86.08)	
T	257 (7.13)	1113 (7.26)	0.078
C	3349 (92.87)	14,215 (92.74)	

**Table 9:** Association of rs12104705 in *GCG* gene and T2DM in Han Chinese.

SNPs	Genotypes	Unadjusted ORs (95% CI)	*p*	Adjusted ORs * (95% CI)	p *
rs12104705	CC	1		1	
	CT	0.993 (0.854–1.155)	0.928	0.961 (0.811–1.137)	0.641
	TT	0.830 (0.405–1.699)	0.610	0.965 (0.450–2.069)	0.928
	TT *vs.* CT + CC	0.831 (0.406–1.700)	0.612	0.970 (0.453–2.079)	0.938
	TT + CT *vs.* CC	0.986 (0.850–1.144)	0.853	0.961 (0.814–1.134)	0.636

* Adjusted for sex, age, anthropometric measurements, TC, TG, HDL-C, and LDL-C.

### 2.5. Interaction of LRP5, TCF7L2, and GCG

From MDR analysis of the interaction among the nine SNPs (including our previous results for rs7903146 and rs290487 in *TCF7L2* (Wang *et al.* [13]), we found 2- to 9-locus model for T2DM; eight models were significant. Overall, the 3-locus model involving rs7903146, rs290487, and rs11196218 had the highest level of testing accuracy (55.30%), and presented the best cross-validation consistency (10/10) (Table 10). Therefore, we chose this model as the best MDR model for T2DM to demonstrate potential gene–gene interaction among these SNPs.

**Table 10:** Best gene-gene interaction models identified by the MDR v1.2.0.

Locus No.	Best Combination	Training Accuracy	Testing Accuracy	Sign Test (*p*)	CV Consistency
2	rs290487 rs11196218	0.5436	0.5346	10 (0.0010)	6/10
3	rs7903146 rs290487 rs11196218	0.5552	0.5530	10 (0.0010)	10/10
4	rs7903146 rs290487 rs11228303 rs11196218	0.5634	0.5462	10 (0.0010)	5/10
5	rs7903146 rs290487 rs7102273 rs4930588 rs11196218	0.5747	0.5517	10 (0.0010)	10/10
6	rs7903146 rs290487 rs7102273 rs4930588 rs11196218 rs12104705	0.5884	0.5411	10 (0.0010)	5/10
7	rs7903146 rs290487 rs7102273 rs12363572 rs4930588 rs11196218 rs12104705	0.6035	0.5481	10 (0.0010)	10/10
8	rs7903146 rs290487 rs7102273 rs11228303 rs12363572 rs4930588 rs11196218 rs12104705	0.6174	0.5473	10 (0.0010)	10/10
9	rs7903146 rs290487 rs3758644 rs7102273 rs11228303 rs12363572 rs4930588 rs11196218 rs12104705	0.6240	0.4000	5 (0.6230)	10/10

Because MDR could not reveal the main effect of the interactions, we used multiplicative logistic regression to analyze the interactions between pairs of SNPs after adjustment for confounders. Significant interactions were found between genotype CC of rs12104705 in *GCG* and AG of rs11196218 in *TCF7L2*, as well as CC of rs12104705 in *GCG* and TT of rs7102273 in *LRP5* (Table 11). However, significant antagonistic interactions were revealed for the genotype CC of rs12104705 in *GCG* and TT of rs4930588 in *LRP5*; CT genotype of rs290487 in *TCF7L2* and CC of rs7903146 in *TCF7L2*; and CC of rs12363572 in *LRP5* and TT of rs7102273 in *LRP5*; and CT of rs12363572 in *LRP5* and TT of rs7102273 in *LRP5*.

**Table 11:** Interactions of SNPs in *LRP5*, *TCF7L2*, and *GCG* gene for T2DM by the multiplicative logistic regression.

SNP		OR (95% CI) *	p *
*GCG* rs12104705	TCF7L2 rs11196218		
CC	AG	2.461 (1.198–5.056)	0.014
*GCG* rs12104705	LRP5 rs7102273		
CC	TT	1.709 (1.008–2.897)	0.047
*GCG* rs12104705	LRP5 rs4930588		
CC	TT	0.672 (0.341–1.326)	0.252
TCF7L2 rs290487	TCF7L2 rs7903146		
CT	CC	0.343 (0.205–0.574)	0.001
TCF7L2 rs290487	LRP5 rs7102273		
CT	TT	0.604 (0.372–0.981)	0.042
LRP5 rs12363572	LRP5 rs7102273		
CC	TT	0.610 (0.399–0.934)	0.023
LRP5 rs12363572	LRP5 rs7102273		
CT	TT	0.330 (0.163–0.667)	0.002

* Adjusted for sex, age, anthropometric measurements, TC, TG, HDL-C, and LDL-C.

## 3. Discussion

The WNT signaling pathway exerts its effect via the 7-transmembrane domain frizzled receptors and *LRP5/6* co-receptors. The key effectors of the WNT signaling pathway is β-catenin/TCF, which is formed by β-catenin and a member of the TCF family (the most common being *TCF7L2*). WNT signals are transmitted to the WNT receptors to prevent the phosphorylation-dependent degradation of β-catenin, and then it enters the nucleus with TCF to form aβ-catenin-TCF complex to regulate downstream target genes such as *GCG* [14]. Furthermore, dominant negative TCF can repress both endogenous and lithium-stimulated WNT-mediated expression of the proglucagon gene in intestinal L cells [15]. The WNT signaling pathway was initially known as a key transduction pathway in a various human cancers and embryonic development [16,17].

We investigated the association of SNPs in the WNT signaling genes *LRP5* and *TCF7L2* and downstream *GCG* and risk of T2DM in Han Chinese: 1842 patients with T2DM and 7777 normal glucose-tolerant healthy participants. Among the five SNPs in *LRP5*, the recessive models of rs7102273 and haplotype GCTCC were associated with risk of T2DM; the haplotype GCTTC was associated with decrease risk. For *TCF7L2*, the rs11196218 genotype GA and haplotypes CCG, TTG, and TTA were associated with increased risk and haplotype CTG and TCG with decreased risk. MDR and multiplicative logistic regression revealed potential gene–gene interactions among *LRP5*, *TCF7L2*, and *GCG* and T2DM. The WNT signaling pathway may play a significant role in risk of T2DM in the Han Chinese population, *LRP5* played an important role in glucose and lipid metabolism in animal experiments [9], and the location of *LRP5* is in the region with T1DM linkage [10]. In addition, in a large family-based genetic association study, polymorphisms of the *LPR5* were associated with adult obesity [18]. However, the associations of SNPs in *LPR5* and T2DM have only been studied in a Japanese population [19], with no association found. In our study, among the five SNPs, only the recessive model of rs7102273 was associated with T2DM, after adjustment for other confounders. The effect of a single SNP is weak for risk of diseases whereas the haplotype block has a major role. We found that the haplotype GCTCC could significantly increase risk of T2DM and GCTTC could decrease the risk.

*TCF7L2* is a specific transcription factor produced by intestinal cells, and *in vitro TCF7L2* knockout experiments showed that increased apoptosis of islet βcells was accompanied by decreased proliferation [20]. Genome-wide association study (GWAS) confirmed that rs7903146 of *TCF7L2* was significantly associated with T2DM in a Caucasian population [21]. The recent data showed that the rs7903146 enhances the expression of TCF4 in pancreatic beta-cell [22] and influence the development of T2DM via regulating the levels of GLP-1 [15,23]. The high level of TCF4 expression can suppress the GLP-1’s expression and the GLP-1 induced insulin secretion [22,24]. Because of the low allele frequency of rs7903146, most previous studies were underpowered to detect an association with T2DM in Han Chinese population and the results were inconsistent. rs290487 [25] and rs11196218 [26] were initially found associated with T2DM in the Chinese populations in Taiwan and Hong Kong but were not further validated in a large sample in Chinese people. Our results suggest that the rs11196218 GA genotype was associated with increased risk of T2DM, not observed in previous studies, and the PARP was 11.73%. Hap-analysis revealed the haplotypes CCG, TTG, and TTA significantly associated with increased risk of T2DM and CTG and TCG associated with decreased risk for the first time in Han Chinese population.

GLP-1, produced by alternative processing of the prohormonal precursor proglucagon, is released form intestinal enteroendocrine cells after feeding as a peptide hormone.GLP-1 plays a key role in promoting glucose-dependent insulin secretion, enhancing peripheral insulin sensitivity, reducing blood glucose levels, and inducing satiety [4,23]. *GCG* as the main gene encoding the GLP-1 is the main downstream gene involved in glucose metabolism and islet cell function regulated by the β-catenin-TCF7L2 complex. The association of *GCG* and T2DM has been investigated only in a Danish population [27], with no association found. We also did not find an association of *GCG* and T2DM.

In addition to the independent function of the three genes in WNT signaling pathway, some studies suggested that polymorphisms of the transmembrane receptor gene *LRP5* in WNT signaling may affect the formation of the β-catenin-*TCF7L2* complex, and the interaction between *LRP5* and *TCF7L2* may affect the expression of downstream regulated genes [24]. No study has confirmed these assumptions. To reduce the type I error probability in analyzing interactions among genes, we used both MDR and multiplicative logistic regression and found potential gene-gene interactions among *LRP5*, *TCF7L2*, and *GCG* associated with T2DM.

Our study is the first to assess the relationships between *LRP5* and *GCG* in the Han Chinese population. As well, we analyzed the interaction between SNPs in the WNT signaling pathway for the first time. All genotypes were in Hardy-Weinberg equilibrium in controls (*p* > 0.05) and the power of our study to detect the association of the significant SNPs in the WNT signaling pathway and T2DM in the Chinese population was 100% by power for genetic association analyses *vs.* sample size package (PGA). Via our research, we screened the useful markers of T2DM for Chinese, and provide the evidence for the primary prevention of T2DM in China. However, limitations should be considered in our study. First, the interactions between the SNPs and the behavior risk factors were not evaluated in our manuscript due to lack of information. Second, some factors like genders and ages were not comparable between the T2DM patients and healthy controls. Third, selection bias may exist between the cases and controls, and our cases were from urban and rural areas, whereas the controls were almost from urban area.

In conclusion, although we found no substantial association of the main SNPs in *LRP5* and *GCG* and T2DM, analysis of the haplotypes revealed that the WNT signaling pathway plays a significant role in risk of T2DM, with interactions among the three genes studied in a Han Chinese population. However, more representative and comprehensive studies in people of different ethnic backgrounds are needed to clarify the mechanisms and underlying genetic effects of T2DM.

## 4. Materials and Methods

### 4.1. Patients and Controls

Participants were of Han Chinese ancestry among local inhabitants of Henan Province. We recruited 1842 patients with T2DM from the outpatient clinics of several hospitals from 2010 to 2011. T2DM was diagnosed in accordance with the American Diabetic Association criteria [28]. We recruited 7777 non-diabetic healthy controls from rural communities in Henan Province in 2008. The sample size was calculated by PGA package (the condition: Disease Prevalence in China, 9.7%; Disease Allele Frequency (the lowest MAF in Chinese among these SNPs), 0.011; α = 0.05; 1-β = 0.75; case to control ratio, 1:4). We excluded participants who were <25 or >75 years old; had body mass index (BMI) <18.5; were pregnant, handicapped, or mentally disturbed; had obesity caused by disease or were taking certain drugs; and had cancer or were unable or unwilling to participate. We obtained informed consent from all participants, and the study was approved by the Ethics Committees of Zhengzhou University.

Data on demographic and anthropometric characteristics were collected by interviewer-administered questionnaire. Anthropometric measurements included body weight, body height, BMI, waist circumference (WC), and blood pressure. An electronic sphygmomanometer was used to measure blood pressure.

### 4.2. Biochemical Measurements

All blood samples were combined with disodium EDTA for measuring glucose level and non-EDTA for measuring total cholesterol (TC), triglyceride (TG), and high-density lipoprotein-cholesterol (HDL-C) by use of an automatic biochemical analysis instrument. Low-density lipoprotein-cholesterol (LDL-C) was calculated by the Freidwald formula [29].

### 4.3. DNA Isolation and SNP Selection

Genomic DNA was extracted from whole blood by use of a blood genome DNA extraction kit (Yaneng BIO, Shenzhen, China). The tagging SNPs were selected for genotyping from the Phase II HapMap Han Chinese in Beijing (CHB) population by use of Haploview 4.2 (www.broad.mit.edu). The final SNPs selected from preliminary experiments are in Table 12. The linkage disequilibrium structures between the SNPs in *LRP5* and *TCF7L2* in the Han Chinese population are in Table 13 and Table 14.

**Table 12:** The final selected SNPs involved in the present study.

Gene	SNPs	Position	Alleles	MAF *
*LRP5*	rs3758644	68,122,664	C/T	0.011
	rs7102273	68,142,155	T/C	0.156
	rs4930588	68,171,811	T/G	0.116
	rs12363572	68,145,542	C/T	0.047
	rs11228303	68,156,745	C/T	0.044
*TCF7L2*	rs11196218	114,830,484	G/A	0.407
*GCG*	rs12104705	162,999,863	C/T	0.066

* MAF: Minor allele frequencies in the Chinese population.

**Table 13:** The linkage disequilibrium (as D’/r2) between SNPs of *LRP5* gene in the Chinese population.

D’/r^2^	rs7102273	rs4930588	rs12363572	rs11228303
rs3759644	0.620/0.002	0.610/0.001	0.734/0.001	0.158/0.000
rs7102273	-	0.749/0.296	0.582/0.089	0.036/0.000
rs4930588	-	-	0.495/0.003	0.002/0.000
rs12363572	-	-	-	0.011/0.000

**Table 14:** The linkage disequilibrium (as D’/r2) between SNPs of *TCF7L2* gene in the Chinese population.

D’/r^2^	rs11196218	rs290487
rs7903146	0.620/0.002	0.610/0.001
rs11196218	-	0.749/0.296

### 4.4. Genotyping

Genotyping involved use of PCR-restriction fragment length polymorphism (PCR-RFLP) and PCR- ligase detection reaction (PCR-LDR). The PCR-RFLP primers were designed by use of Primer Premier v5.0 (PREMIER Biosoft International, Palo Alto, CA, USA) and the sequences of PCR primers and conditions for RFLP are in Table 15. PCR was carried out in a 20 μL reaction volume containing 50 ng genomic DNA, 5 pmoL each primer, 10 μL 2 × Taq PCR mix ([Laifeng BIO, Shanghai, China], containing 1 mmoL/L of MgCl2, 100 μmmoL/L of deoxynucleotide triphospate (dNTP), and 0.5U of Taq polymerase). PCR products were incubated for 10 h with 3U restriction enzyme in a 20-μL reaction volume and separated by 4% agarose gel electrophoresis. To verify the reproducibility, we repeated 5% samples at random as a quality controls for genotyping, and the concordance rate was 99%. rs11196218 in *TCF7L2* and rs12104705 in *GCG* were genotyped by use of PCF-LDR by Shanghai Generay Biotech.

**Table 15:** Primer sequences and restriction enzymes for PCR-RFLP.

Gene	Polymorphisms	Primer sequences	Restriction Enzymes (T)
*LRP5*	rs3758644	5′-GATGAGCTCCTCAGAGTCCGTG-3′ (F)	BseNI (Fermantas), (65 °C)
		5′-GGATGAGGTTCGCGTTTACCTA-3′ (R)	
	rs7102273	5′-AAGCATTGTGAGGGAGAACACC-3′ (F)	PaeI (Fermantas), (37 °C)
		5′-GTGTCTAACCCAGGGATGCAGA-3′ (R)	
	rs4930588	5′-TGGCCTTTAATTGCCTGCACCAG-3′ (F)	BseNI (Fermantas), (65 °C)
		5′-CAAAGCAGAGTTGGAACGGACT-3′ (R)	
	rs12363572	5′-AGTGGGAAGATCCCTTGAGTCC-3′ (F)	TaiI (Fermantas), (65 °C)
		5′-TCTATTCACGTCCTTTGCCCAT-3′ (R)	
	rs11228303	5′-CTACCCAAATCCTATAAA-3′ (F)	HinfI (Fermantas), (37 °C)
		5′-GGGCTATGAGCTAGTT AAG-3′ (R)	

## 5. Statistical Analysis

Categorical data are shown as number (percentage) and analyzed by chi-square test for. Continuous data are shown as median (range) for data with non-normal distribution. Mann-Whitney-Wilcoxon and Kruskal-Wallis rank tests were used to assess differences between cases and controls. Hardy-Weinberg equilibrium for each SNP was calculated for cases and controls by use of Haploview 4.2. Odds ratios (ORs), 95% confidence intervals (95% CIs) and corresponding *p*-values for risk of T2DM were calculated by logistic regression analyses after adjusting for sex, age, anthropometric measurements (BMI, WC, and blood pressure), and biochemical indexes (lipid levels including TC, TG, HDL-C and LDL-C). The interactions among SNPs for the three genes were analyzed by multifactor dimensionality reduction (MDR) and multiplicative logistic regression after adjusting for sex, age, and BMI. All tests were two-sided and were considered statistically significant at *p* ≤ 0.05. Calculation of haplotype and linkage disequilibrium coefficients involved use of Haploview 4.2 (www.broad.mit.edu). *P* ≤ 0.05 was considered statistically significant. Calculation of PARP was based on the estimated ORs and genotypic frequencies of the SNPs showing significant association with T2DM. All statistical analyses involved use of SPSS v17.0 for Windows (SPSS Inc., Chicago, IL, USA). Power calculation involved use of power for PGA package [30].

## References

[1] American Diabetes Association (2011). Diagnosis and classification of diabetes mellitus. Diabetes Care.

[2] Huelgas R.G. (2012). A clinical trial to maintain glycemic control in youth with type 2 diabetes. Rev. Clin. Esp..

[3] Palsgaard J., Emanuelli B., Winnay J.N., Sumara G., Karsenty G., Kahn C.R. (2012). Cross-talk between insulin and Wnt signaling in preadipocytes: Role of Wnt co-receptor low density lipoprotein receptor-related protein-5 (LRP5). J. Biol. Chem..

[4] Liu Z., Habener J.F. (2008). Glucagon-like peptide-1 activation of TCF7L2-dependent Wnt signaling enhances pancreatic beta cell proliferation. J. Biol. Chem..

[5] Rulifson I.C., Karnik S.K., Heiser P.W., Ten B.D., Chen H., Gu X., Taketo M.M., Nusse R., Hebrok M., Kim S.K. (2007). Wnt signaling regulates pancreatic beta cell proliferation. Proc. Natl. Acad. Sci. USA.

[6] Fujino T., Asaba H., Kang M.J., Ikeda Y., Sone H., Takada S., Kim D.H., Ioka R.X., Ono M., Tomoyori H. (2003). Low-density lipoprotein receptor-related protein 5 (LRP5) is essential for normal cholesterol metabolism and glucose-induced insulin secretion. Proc. Natl. Acad. Sci. USA.

[7] Schinner S. (2009). Wnt-signalling and the metabolic syndrome. Horm. Metab. Res..

[8] Logan C.Y., Nusse R. (2004). The Wnt signaling pathway in development and disease. Annu. Rev. Cell. Dev. Biol..

[9] Schinner S., Ulgen F., Papewalis C., Schott M., Woelk A., Vidal-Puig A., Scherbaum W.A. (2008). Regulation of insulin secretion, glucokinase gene transcription and beta cell proliferation by adipocyte-derived Wnt signalling molecules. Diabetologia.

[10] Twells R.C., Mein C.A., Payne F., Veijola R., Gilbey M., Bright M., Timms A., Nakagawa Y., Snook H., Nutland S. (2003). Linkage and association mapping of the LRP5 locus on chromosome 11q13 in type 1 diabetes. Hum. Genet..

[11] Peng S., Zhu Y., Lu B., Xu F., Li X., Lai M. (2013). TCF7L2 gene polymorphisms and type 2 diabetes risk: A comprehensive and updated meta-analysis involving 121 174 subjects. Mutagenesis.

[12] Willard F.S., Sloop K.W. (2012). Physiology and emerging biochemistry of the glucagon-like peptide-1 receptor. Exp. Diabetes Res..

[13] Wang J., Li L., Zhang J., Xie J., Luo X., Yu D., Zhao J., Feng T., Pang C., Yin L., Hu F., Zhang J., Wang Y., Wang Q., Zhai Y., You H., Zhu T., Hu D. (2013). Association of rs7903146 (IVS3C/T) and rs290487 (IVS3C/T) polymorphisms in TCF7L2 with type 2 diabetes in 9,619 Han Chinese population. PLoS ONE.

[14] Zeng X., Tamai K., Doble B., Li S., Huang H., Habas R., Okamura H., Woodgett J., He X. (2005). A dual-kinase mechanism for Wnt co-receptor phosphorylation and activation. Nature.

[15] Yi F., Brubaker P.L., Jin T. (2005). TCF-4 mediates cell type-specific regulation of proglucagon gene expression by beta-catenin and glycogen synthase kinase-3beta. J. Biol. Chem..

[16] Peifer M., Polakis P. (2000). Wnt signaling in oncogenesis and embryogenesis—A look outside the nucleus. Science.

[17] Moon R.T., Brown J.D., Torres M. (1997). WNTs modulate cell fate and behavior during vertebrate development. Trends Genet..

[18] Guo Y.F., Xiong D.H., Shen H., Zhao L.J., Xiao P., Guo Y., Wang W., Yang T.L., Recker R.R., Deng H.W. (2006). Polymorphisms of the low-density lipoprotein receptor-related protein 5 (LRP5) gene are associated with obesity phenotypes in a large family-based association study. J. Med. Genet..

[19] Zenibayashi M., Miyake K., Horikawa Y., Hirota Y., Teranishi T., Kouyama K., Sakaguchi K., Takeda J., Kasuga M. (2008). Lack of association of LRP5 and LRP6 polymorphisms with type 2 diabetes mellitus in the Japanese population. Endocr. J..

[20] Cauchi S., Meyre D., Dina C., Choquet H., Samson C., Gallina S., Balkau B., Charpentier G., Pattou F., Stetsyuk V. (2006). Transcription factor TCF7L2 genetic study in the French population: Expression in human beta-cells and adipose tissue and strong association with type 2 diabetes. Diabetes.

[21] Florez J.C., Jablonski K.A., Bayley N., Pollin T.I., de Bakker P.I., Shuldiner A.R., Knowler W.C., Nathan D.M., Altshuler D. (2006). TCF7L2 polymorphisms and progression to diabetes in the Diabetes Prevention Program. N. Engl. J. Med..

[22] Cauchi S., Froguel P. (2008). TCF7L2 genetic defect and type 2 diabetes. Curr. Diabetes Rep..

[23] Yi F., Sun J., Lim G.E., Fantus I.G., Brubaker P.L., Jin T. (2008). Cross talk between the insulin and Wnt signaling pathways: Evidence from intestinal endocrine L cells. Endocrinology.

[24] Lyssenko V., Lupi R., Marchetti P., Del G.S., Orho-Melander M., Almgren P., Sjogren M., Ling C., Eriksson K.F., Lethagen A.L. (2007). Mechanisms by which common variants in the TCF7L2 gene increase risk of type 2 diabetes. J. Clin. Investig..

[25] Chang Y.C., Chang T.J., Jiang Y.D., Kuo S.S., Lee K.C., Chiu K.C., Chuang L.M. (2007). Association study of the genetic polymorphisms of the transcription factor 7-like 2 (TCF7L2) gene and type 2 diabetes in the Chinese population. Diabetes.

[26] Ng M.C., Tam C.H., Lam V.K., So W.Y., Ma R.C., Chan J.C. (2007). Replication and identification of novel variants at TCF7L2 associated with type 2 diabetes in Hong Kong Chinese. J. Clin. Endocrinol. Metab..

[27] Torekov S.S., Ma L., Grarup N., Hartmann B., Hainerova I.A., Kielgast U., Kissow H., Rosenkilde M., Lebl J., Witte D.R.. (2011). Homozygous carriers of the G allele of rs4664447 of the glucagon gene (GCG) are characterised by decreased fasting and stimulated levels of insulin, glucagon and glucagon-like peptide (GLP)-1. Diabetologia.

[28] American Diabetes Association (2005). Standards of medical care in diabetes. Diabetes Care.

[29] Cantin B., Lamarche B., Despres J.P., Dagenais G.R. (2002). Does correction of the friedewald formula using lipoprotein(a) change our estimation of ischemic heart disease risk? The Quebec Cardiovascular Study. Atherosclerosis.

[30] National Cancer Institute Power for Genetic Association Analyses (PGA). http://dceg.cancer.gov/bb/tools/pga.

